# The Effect of Probiotic Supplementation on the Gut–Brain Axis in Psychiatric Patients

**DOI:** 10.3390/cimb45050260

**Published:** 2023-05-06

**Authors:** Hussein Sabit, Areej Kassab, Donia Alaa, Shaza Mohamed, Shaimaa Abdel-Ghany, Mohamed Mansy, Osama A. Said, Mona A. Khalifa, Halah Hafiz, Asmaa M. Abushady

**Affiliations:** 1 Department of Medical Biotechnology, College of Biotechnology, Misr University for Science and Technology, Giza P.O. Box 77, Egypt; 2 Department of Environmental Biotechnology, College of Biotechnology, Misr University for Science and Technology, Giza P.O. Box 77, Egypt; 3 Department of Maxillofacial Surgery and Diagnostic Sciences, College of Dentistry, Jazan University, Jazan 45142, Saudi Arabia; 4 Department of Agricultural Biotechnology, College of Biotechnology, Misr University for Science and Technology, Giza P.O. Box 77, Egypt; 5 Faculty of Art and Science, Samtah, Jazan University, Jazan 45142, Saudi Arabia; 6 Clinical Nutrition Department, Factually of Applied Medical Science, Umm Alqura University, Mecca 24382, Saudi Arabia; 7 School of Biotechnology, Nile University, Giza 41516, Egypt; 8 Genetic Department, Faculty of Agriculture, Ain Shams University, Cairo 11566, Egypt

**Keywords:** psychiatric disorders, gut microbiota, gut–brain axis, mouth–gut axis, probiotics

## Abstract

The pathophysiology of several psychiatric diseases may entail disturbances in the hypothalamic–pituitary–adrenal (HPA) axis and metabolic pathways. Variations in how these effects present themselves may be connected to individual variances in clinical symptoms and treatment responses, such as the observation that a significant fraction of participants do not respond to current antipsychotic drugs. A bidirectional signaling pathway between the central nervous system and the gastrointestinal tract is known as the microbiota–gut–brain axis. The large and small intestines contain more than 100 trillion microbial cells, contributing to the intestinal ecosystem’s incredible complexity. Interactions between the microbiota and intestinal epithelium can alter brain physiology and affect mood and behavior. There has recently been a focus on how these relationships impact mental health. According to evidence, intestinal microbiota may play a role in neurological and mental illnesses. Intestinal metabolites of microbial origin, such as short-chain fatty acids, tryptophan metabolites, and bacterial components that might stimulate the host’s immune system, are mentioned in this review. We aim to shed some on the growing role of gut microbiota in inducing/manipulating several psychiatric disorders, which may pave the way for novel microbiota-based therapies.

## 1. Introduction

Mental disorders are among the leading causes of nonfatal disease burden around the world, though with severe consequences [1]. Over a third of the EU population suffers from mental disorders annually. The true prevalence of “brain disorders”, including neurological disorders, is significantly higher. Brain diseases, as the central health concern of the twenty-first century, necessitate comprehensive priority action at all levels, including significantly increased funding for scientific, clinical, and public health research to develop better techniques for improved prevention and treatment [2]. Anxiety disorders, bipolar disorder, schizophrenia, autism spectrum disorders, conduct disorder, attention deficit hyperactivity disorder, eating disorders, idiopathic developmental intellectual disability, and a broad category of other mental diseases are examples of these disorders [3]. Given the large number of people in need and the imperative to alleviate suffering, it is urgent to implement scalable mental health interventions to alleviate this burden [3].

Over thousands of years, the host and the “gut microbiota”, which includes bacteria, archaea, and eukaryotes that inhabit the gastrointestinal (GI) tract, have co-evolved to create a complex and beneficial connection [4]. The microbiota serves the host in several ways via various physiological functions, such as maintaining gut integrity and epithelial shape, gathering energy, preventing pathogens, and controlling host immunity. However, dysbiosis, which is defined by a shift in microbial composition, has the potential to disrupt these processes [5].

According to estimates, more than 10^14^ microorganisms reside in the GI tract, with bacterial cells outnumbering human cells by a factor of ten and the microbiome’s genetic material outnumbering the human genome by a factor of more than 100 [6]. The gut microbiota is a crucial player in the regulation of the gut–brain axis because it can secrete and upregulate vital proteins and metabolites involved in the release of gut hormones and neuropeptides, such as short-chain fatty acids, and control the synthesis of neurotransmitters and their precursors (such as serotonin, γ-aminobutyric acid (GABA), and tryptophan) [7].

Trillions of bacteria inhabit the human gut, and it has been shown that these bacteria are crucial for gut–brain communication via regulating endocrine, immunological, and neurological pathways. Patients with various mental illnesses, such as depression, bipolar disorder, schizophrenia, and autism spectrum disorder, have been shown to have drastically different gut microbiomes [8].

Probiotics, prebiotics, and dietary modifications that increase the number of good bacteria in the gut may improve mood and lessen anxiety and depression. They should be employed instead of traditional medicine due to its side effects [9].

The mechanisms of the gut–brain connection include neuro–immuno–endocrine mediators. This bidirectional neurological link includes the ENS, the autonomic nervous system (ANS), the central nervous system (CNS), the brain and spinal cord, and the hypothalamic–pituitary–adrenal (HPA) axis. The ANS, sympathetic and parasympathetic limbs control afferent signals coming from the lumen and traveling to the CNS through enteric, spinal, and vagal pathways, as well as efferent signals traveling from the CNS to the intestinal wall. The HPA axis coordinates organisms’ adaptive responses to many stimuli, making it the primary stress–efferent axis [10].

This system is activated by environmental stress and increased levels of systemic pro-inflammatory cytokines. Adrenocorticotropic hormone secretion from the pituitary gland is then activated, which causes the release of corticotropin-releasing factor from the hypothalamus, which in turn causes the release of cortisol from the adrenal glands [11].

The present review describes the main composition of gut microbiota and their metabolites that affect several psychiatric disorders.

## 2. Probiotics

Elie Metchnikoff presented the concept of probiotics to the scientific community for the first time [12]. According to the Food and Agriculture Organization of the United Nations (FAO) and the World Health Organization (WHO), probiotics are described as “living bacteria that, when ingested in suitable concentrations, confer health advantages on the host” [13]. A minimum of 10^6^–10^7^ CFU/g of live probiotics is required for health benefits to be noticed, despite the absence of reliable information regarding minimum effective concentrations [14]. Probiotics should be able to improve human health and sustain well-being on the one hand. On the other hand, long-term and possibly chronic effects should be included in the safety assessment [15]. 

Mechanisms of probiotics include colonization and normalization of disturbed intestinal microbial communities in children and adults [16]. 

Though it is not the focus of the present review, we must mention that Gram-negative bacteria were also used as probiotics. Since its discovery by the army surgeon Alfred Nissle in 1917, *E. coli* has been used to treat some GIT disorders, such as constipation [17] and colitis [18]. This theory is supported by the long medical history of *E. coli* as a microbial remedy in Central Europe and by the extensive trials that have demonstrated its probiotic effect [19]. Since then, *E. coli* has been used in different microbial-based remedies worldwide [18].

## 3. Probiotic Bacteria

**Figure 1:**
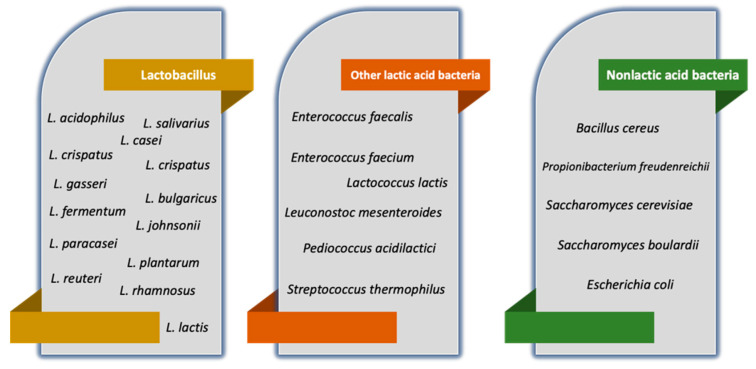
Microorganisms that are considered probiotics. The list contains, but is not limited to, *lactobacillus* and non-lactic acid bacteria.

*Lactobacillus* spp., *Bifidobacterium* spp., and *Enterococcus* spp. are the most used probiotic microorganisms in human nutrition; whereas in ruminants, yeast, particularly Saccharomyces cerevisiae, plays a significant role; whereas in pigs and poultry, *Bacillus* spp., *Enterococcus* spp., and *Lactobacillus* spp. However, it is noteworthy that the health advantages of probiotics are strain-specific, not species- or genus-specific [15]. Figure 1 represents the most common microorganisms used as probiotics.

## 4. Sources of Probiotics

Probiotics are usually found in fermented dairy products such as yogurt, cultured buttermilk, and cheese. Additional sources of bacterial fermentation include Japanese miso, tempeh, sauerkraut, beer, sourdough, bread, chocolate, kimchi, kefir, olives, and pickles. However, yogurts and fermented milk remain probiotics’ most prevalent food transporter, as it has been demonstrated that nondairy fermented substrates, such as soy-based products, cereals, legumes, cabbage, maize, pearl millet, and sorghum, contain probiotic bacteria [20].

## 5. Health-Related Issues of Probiotics

Probiotics have been demonstrated to impact various physiological states and diseases. They protect against intestinal disorders such as antibiotic-associated diarrhea, traveler’s diarrhea, irritable bowel syndrome, and inflammatory bowel disease. Secondly, they have immunomodulation properties by enhancing allergy resistance, boosting innate immunity, and preventing respiratory illnesses. Thirdly, they have medicinal properties such as preventing urogenital infections, producing vitamins B2, B6, and B12, preventing rotaviral diarrhea, and treating skin and oral illnesses. They have metabolic benefits such as lactose hydrolase, which improves lactose digestion; bile salt deconjugation, which reduces cholesterol; mutagenic reaction suppression in the stomach, which promotes anti-carcinogenic activity; and calcium metabolism augmentation, which prevents osteoporosis [20]. In addition, regulation of the gut–brain by probiotics has been proposed as an innovative treatment for anxiety and depression [21].

The FDA’s standards do not address probiotics’ risks, such as the potential to introduce new genes into consumers’ microbiomes. Particularly problematic are antibiotic resistance genes, as probiotic bacteria may have innate or acquired antibiotic resistance like other bacteria. However, only one study involving seven subjects has investigated the likelihood of such transfers occurring in the human intestine [22]. 

In addition, the inherent infectious properties of the bacteria contained in probiotic supplements may constitute a risk to specific individuals. Few case reports have indicated severe side effects, particularly in immunocompromised patients, including fungal and bacterial infections. Due to poorly organized reporting of adverse events in most probiotic clinical trials and a lack of reliable processes to identify post-marketing harm from supplements, the exact rates of opportunistic infections associated with probiotic supplements are unknown [23].

**Figure 2:**
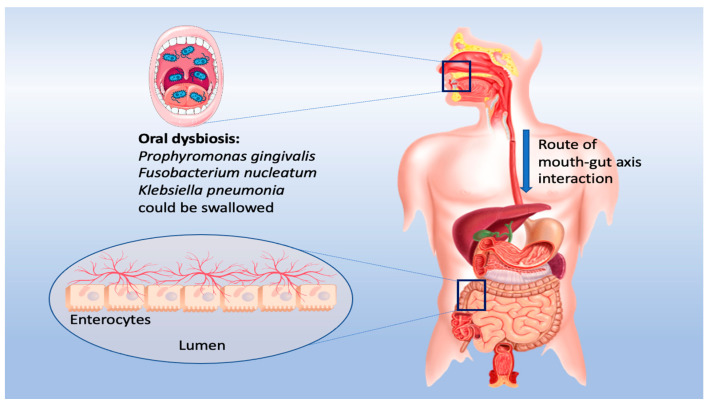
The proposed mouth–gut axis.

Meanwhile, oral microbiota also plays a crucial role in health and disease, where the oral cavity and gut are the two largest microbial habitats and play a significant role in microbiome-related disorders (Figure 2). Even though the mouth cavity and gut are continuous regions connected by the gastrointestinal system, their microbiome profiles are well-separated due to the oral–gut barrier, physical distance, and chemical barriers, such as stomach acid and bile [24,25]. 

However, when the mouth–gut barrier is compromised, oral microbiota can be translocated to the gut. In general, infants and the elderly have immature or less practical bodily barriers [26,27]. Intriguingly, gut-dwelling *Bifidobacterium* has been found in infants’ oral fluid [28]. Similarly, aged people have a higher incidence of oral bacteria in the gut than healthy adults, including *Porphyromonas*, *Fusobacterium*, and *Pseudoramibacter* [29,30]. Oral microbiota can penetrate the gut and change the gut microbial population, as revealed in vitro by Li and his colleagues [31].

Typical oral-dwelling species have been found in the GI tract under diseased situations [32]. For instance, in the gut mucosa of patients with inflammatory bowel disease (IBD), the oral commensal bacteria *Haemophilus* and *Veillonella* were significantly enriched [33]. Several oral taxa, including *Fusobacterium*, were detected in the gut microbiomes of colon cancer patients [34]. This means that the average human oral microbiota can penetrate and colonize the gut mucosa and become an opportunistic pathogen in the presence of mucosal homeostasis disruption [35].

Similarly, enteric bacteria can spread to the oral cavity through intrapersonal fecal–oral pathways via direct contact or indirect exposure via contaminated fluids and meals [36]. This occurs due to a lack of clean water supply and public health system [37,38] and/or immunocompromised conditions highly related to oral colonization of gram-negative enteric rods, which can be aggravated by poor oral hygiene [39,40].

## 6. Clinical Uses of Probiotics

The most substantial supporting evidence for using probiotics pertains to treating acute diarrhea and pouchitis. Other diseases, such as atopic eczema and genitourinary infections, are among non-GI conditions in which probiotics may have beneficial effects [41]. Hashimoto’s thyroiditis (HT) and Graves’ disease (GD) are other examples [42], though depending on the composition of a patient’s gut bacteria, multifactorial therapeutic and preventive management strategies could be devised and tailored to the individual patient. Future large-scale human studies are required to evaluate the effect of changes in gut microbiota on thyroid function and diseases. Changes in the diversity and composition of microorganisms are increasingly linked to obesity and behavioral disorders, among other conditions, and probiotics have been shown to be helpful in treating these diseases. Energy harvesting, insulin resistance, inflammation, and fat deposition are all influenced by the microbiota that is common in obese people. The intestinal microbiota also plays a significant role in obesity by modulating a variety of metabolic and adipose processes as well as homeostasis, energy balance, central appetite, and reward signaling. Vagal stimulation or immune–neuroendocrine processes may also be used by certain bacterial strains and their metabolites to target the brain. As a result, the gut microbiota has been the target of a number of promising novel anti-obesity medicines [43].

Potential biological mechanisms connecting mental diseases include inflammation, oxidative stress, the gut microbiome, epigenetic changes, and neuroplasticity. Epidemiological studies have shown a correlation between dietary quality measures and psychological well-being across multiple populations and age groups, with the correlation being particularly strong for depression. This correlation cannot be accounted for by any other demographic, lifestyle, or reverse causal factors [44].

Nevertheless, many published studies in the field are preclinical, and clinical studies are scarce. Preliminary studies in psychiatric populations support the notion of dysbiosis in certain conditions; however, these studies are frequently of limited scope and susceptible to confounding variables. There are currently no well-conducted studies on clinical populations, but there are a few on healthy volunteers [45].

## 7. The Immunomodulatory Role of Probiotics

### 7.1. Innate Immune System

Probiotic strains that stimulate the innate immune system include *Bifidobacterium infantis* [46], *B. infantis* 14.518 in Albino BALB/C mice [47], *B. longum*, *B. infantis*, *L. rhamnosus* JB-1 [46], *L. casei*, *B. longum bv*. *infantis* CCUG [48], *Bacillus subtilis* [49], *B. infantis* [50], *L. acidophilus* La1 [15] and *Lactobacillus casei shirota* (LcS) [51]. Multiple probiotic strains of the genus *Bifidobacterium*, including *B. infantis*, *B. adolescentis*, *B. bifidum*, and *B. longum*, may also influence the apoptotic process in intestinal epithelial cells. In addition, they can increase mucin secretion, which is the first line of defense against pathogens in the intestine [52]. Mucin synthesis in intestinal epithelial cells is stimulated by *L. rhamnosus* through the activation of the Muc2 and p40 genes. When an antigen binds to enterocytes, pro-inflammatory neurotransmitters, chemokines, and a small amount of tumor necrosis factor are produced, initiating an effective immune response [53]. 

**Figure 3:**
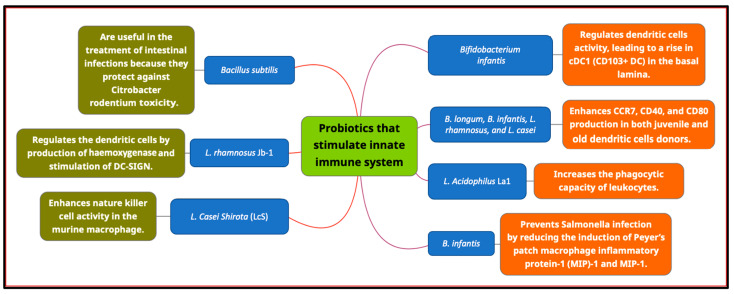
The probiotic bacteria that can stimulate the innate immune system.

After *Clostridium difficile* infection, *L. casei* and *L. rhamnosus* inhibit the generation of pro-inflammatory cytokines in enterocytes. Similarly, after illness, *B. polyfermenticus*, *B. lactus*, *B. animalis* ssp. *lactis*, *L. casei*, *L. paracasei* ssp. *paracasei*, and *L. plantarum* induce the production of natural killer cells [46]. In addition, *L. plantarum* caused murine splenic dendritic cells (DCs) to produce IL-12 [54]. In vitro stimulation of human DCs with particular *Escherichia coli* lipopolysaccharides (LPS) lowered pro-inflammatory cytokines such as IL-2 and TNF-(tumor necrosis factor-alpha) and raised anti-inflammatory cytokines [55]. Single-stranded RNA (ssRNA) from *pediococcus acidilactici* k15 increased IL-10 production in murine DCs [56]. Figure 3 depicts the probiotic bacteria that have a role in the innate immune system.

### 7.2. Humoral Immune System

The goal of probiotics is to make the body more resistant to diseases that could be detrimental. Increased egg albumin-specific humoral immune response was seen after oral administration of *B. bifidum*, while IgA exposure to cholera toxin was enhanced after administration of the same microorganism [57]. Furthermore, *B. breve* suppresses T cell-dependent intestinal inflammation via T-derived IL-10 in B. breve-treated severe combined immunodeficient (SCID) mice [15]. In control experiments, oral administration of *L. rhamnosus* induced IgA-secreting B-cells in children with rotavirus infection [58]. Consumption of fermented milk containing *B. bifidum* and *L. acidophilus* La1 following immunization against Salmonella typhi Ty21 has significantly increased IgA serum content. Another study indicated that a peptide fraction produced from *L. helveticus*-fermented milk induced local mucosal and systemic IgA immune responses in *E. coli* O157:H7-infected mice [15]. 

**Figure 4:**
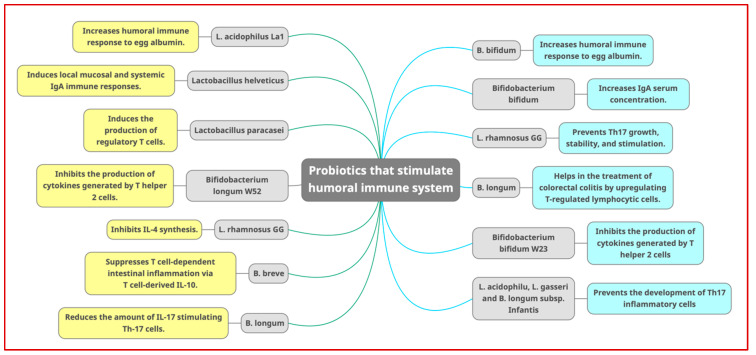
The probiotic bacteria that can stimulate the humoral immune system. B in this figure in all sites is for *Bifidobacterium*.

The number of cells that secrete lactoglobulin antibodies increased after oral administration of *Lactobacilli* to rats sensitized to cow’s milk. Atopic dermatitis develops in infants who consume cow’s milk. On the other hand, probiotic therapy has been scientifically demonstrated to reduce atopic dermatitis infection in humans [46]. Figure 4 illustrates the probiotic bacteria that have a role in the humoral immune system.

It has been demonstrated that probiotic-derived proteases may digest cow milk casein and create peptides that decrease inflammatory cytokines in healthy individuals. A study was performed to determine whether caseins degraded by probiotic bacteria-producing proteases could stimulate the generation of cytokine and anti-CD3 immunoglobulin mononuclear cells in infants with cow milk allergy-associated atopic dermatitis. Casein from cow’s milk promotes IL-4 production, resulting in hypersensitivity [59]. In contrast, oral dosing of *L. rhamnosus* GG degrades casein and suppresses IL-4 production. These findings suggest that probiotics in the diet affect the makeup of potentially dangerous microorganisms, altering their immunogenicity [60].

*Lactobacillus paracasei* induces the development of regulatory T cells, which decrease the effector responses of T helper 1 and 2 cells. By upregulating T-regulated lymphocytic cells, *B. longum* has aided in treating colorectal colitis in mice. As a result, blood levels of IL-10 and IL-12 have increased, whereas inflammatory cytokines such as IL-23, IL-12, and IL-27 have dropped [61]. Through the generation of IL-10 by monocytes, *B. bifidum* W23 and *B. longum* W52 limit the production of cytokines by T helper 2 cells [15]. *B. infantis* stimulates the activation of Foxp3 T-cells in healthy individuals, which reduces the levels of inflammatory cytokines in people with psoriasis [46].

The probiotic strain that produced short-chain fatty acid (SCFA) molecules such as propionate, isobutyrate, acetate, butyrate, etc., directly or indirectly affecting T-cells’ homeostasis. Butyrate stimulates the development of Foxp3+ cells and Treg cells outside the hypothalamus. Propionate controlled T-cell production by blocking histone deacetylase. Probiotics include *L. acidophilus*, *B. breve*, *L. gasseri*, *B. longum*, and *B. longum subsp*. *infantis*, inhibited the production of Th17 inflammatory cells, which are essential for the pathophysiology and progression of various inflammatory illnesses, including irritable bowel syndrome [62]. In addition, *L. rhamnosus* GG and B. breve block IL-17 and IL-23, which are essential for the proliferation, stability, and activation of Th17. Various *Lactobacillus* and *Bifidobacterium* species produced INF and TNF-ɑ, which prevented the growth of Th17 inflammatory cells. *B. longum* promoted the production of IL-27, which has been associated with a decrease in the quantity of IL-17 activating Th-17 cells [46].

## 8. Gut Microbiota

### 8.1. The Gut Microbiota

The term gut microbiota refers to the community of microorganisms that inhabit the gut lumen. In addition to fungi, viruses, and archaea, the adult gut microbiota consists of 10^13^ bacterial cells from more than 250 different species of bacteria [63]. The bulk of gut bacteria are *Firmicutes* (60–80%), *Bacteroidetes* (20–40%), *Proteobacteria*, and *Actinobacteria*, but their relative abundances vary significantly between individuals and depend on anatomical location [64].

### 8.2. Gut Microbiota Metabolites

**Figure 5:**
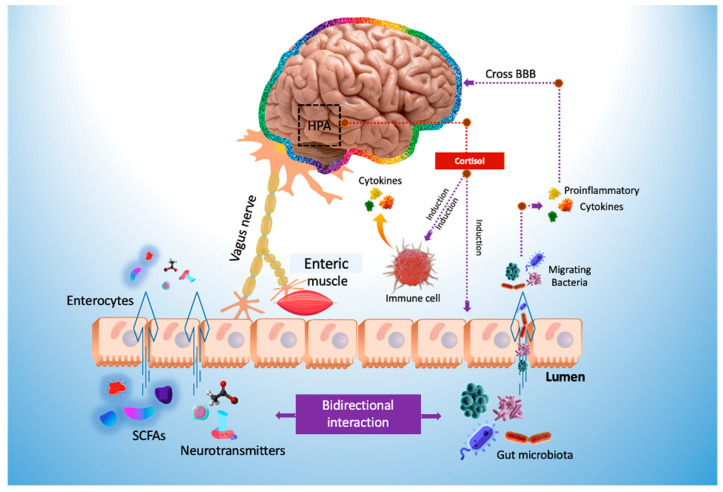
Modulation of the gut–brain axis by probiotics. HPA axis, hypothalamus–pituitary gland–adrenal gland axis; SCFAs, short-chain fatty acids.

Microbial metabolites can be found in several biological excretions, such as feces, urine, serum, and cerebrospinal fluid, and tissues, such as the liver and guts, which significantly affect the host’s physiology [65]. Microorganisms can influence central nervous system (CNS) processes and cognitive functioning [66] and play an essential role in modulating stress-related behaviors such as anxiety and depression [67]. Bidirectional communication between the gut and brain can occur via the vagus nerve (Figure 5) or through modulation of the immune system, the hypothalamic–pituitary–adrenal (HPA) axis, and tryptophan metabolism, as well as the gut microbiota’s abilities to generate several neurotransmitters [68,69] or metabolites with neuroactive properties, such as SCFA [70].

SCFAs are among the most extensively studied metabolites produced by gut bacteria. It includes acetate, propionate, and butyrate, which are generated by bacterial fermentation of fibers (e.g., resistant starch, simple sugars, and polysaccharides). SCFAs regulate the metabolism of lipids, cholesterol, and glucose, in addition to anti-inflammatory and immunological responses and the integrity of the intestinal barrier [71].

SCFAs have been shown as a crucial mechanism of the gut–brain connection. As it promotes the production of numerous hormones and neurotransmitters, such as GABA and serotonin, in the gut, it may interact with enteroendocrine cells and augment vagal or systemic circulation-mediated indirect signals to the brain. The capacity of SCFAs to cross the blood-brain barrier (BBB) positively influences its integrity. It stimulates several brain pathways, regulating the amounts of neurotrophic factors, neurotransmitters, and neurogenesis and lowering neuroinflammation and glial dysfunction [72].

Furthermore, alterations in the gut microbiota have been linked to post-traumatic stress disorder (PTSD) by several lines of evidence. However, it is unclear if and how the gut microbiota affects a person’s propensity to develop PTSD. Furthermore, elevated levels of p-cresol were found in the prefrontal cortex of susceptible mice. Mice with this vulnerability also exhibited elevated levels of dopamine and DOPAC in the prefrontal cortex, as well as an unfavorable increase in dopamine D3 receptor expression [73].

Many gut-resident microbes and the diverse array of bacteria found in fermented foods express genetic machinery that enables the synthesis and metabolism of numerous vitamins, including vitamin B1 (thiamine), B2 (riboflavin), B3 (niacin), B5 (pantothenic acid), B6 (pyridoxine), B7 (biotin), B9 (folate), B12 (cobalamin), vitamin K2 (menaquinone), and vitamin A. Therefore, gut microbiota and probiotics rich in bacteria are vital vitamin B sources for the human body. B vitamins are coenzymes in numerous key biochemical activities, including the metabolism of neurotransmitters. They participate in myelin formation, neuroprotection, mitochondrial activities, energy production, and cellular respiration and exert anti-inflammatory and antioxidant actions [74]. The gut microbiota produces distinct metabolites that target the good bacteria and host cells by utilizing the various nutrients and components absorbed in the diet. 

SCFAs are one of the most thoroughly researched compounds originating from gut microbiota. Three primary SCFAs exist: butyrate, propionate, and acetate. Virtually all cell types include at least one SCFA receptor, such as free fatty acid receptor-2 and -3 (FFAR2 and FFAR3) and G-Protein-Coupled Receptor (GPCR) receptors, such as GPR43, GPR41, GPR109a, and Olfr78. Multiple local actions of SCFAs in the gut help maintain intestinal integrity by regulating luminal pH, mucus production, epithelial cell activity, and the immune system. SCFAs are also essential for microbial communication, influencing quorum sensing and preventing the invasion or growth of various microorganisms and pathogens. Prior research has demonstrated the antidepressant impact of butyrate by correcting anhedonia, low energy, and cognitive and social capacities in mice models [75].

Lactate is a crucial metabolite generated by various types of microorganisms, including lactic acid bacteria, *bifidobacteria*, and *proteobacteria*, and it is frequently transformed into SCFA. Due to this, lactate is not abundant in the colon; however, it has been demonstrated that under physiological conditions, this metabolite can cross the BBB to match the brain’s energy requirements, influencing numerous neuronal functions, including excitability, plasticity, and memory consolidation. The gut microbiota is not the sole source of lactate, as this molecule is also created by astrocytes in the brain, which serve as a local reservoir for lactate and transmit it to neurons and, more significantly, muscle cells during exercise [76]. 

Tryptophan (Trp) is a critical amino acid with numerous bioactive effects in the body, mainly via its various metabolites. The essential Trp metabolites for the MGB axis are (a) the transformation of Trp into the neurotransmitter serotonin, which has beneficial effects on brain and gut function, and (b) the metabolism of Trp into kynurenine, tryptamine, and indole, which have neuroendocrine and immune-modulatory effects. Five bacterial phyla, including *Firmicutes*, *Bacteroidetes*, *Actinobacteria*, *Proteobacteria*, and *Fusobacteria* have been linked to Trp metabolism. The formulation with possible psychological advantages contains two psychobiotic (probiotic with mental health benefits) strains: *L. helveticus* R0052 and *B. longum* R0175 [77]. The supplementation of *L. helveticus* R0052 and *B. longum* R0175 lowered anxiety and depression symptoms in research with healthy volunteers. However, no study has studied the effect of this psychobiotic combination on the mental and physical health of MDD patients [78]. 

The formulation with possible psychological advantages consists of two psychobiotic (probiotic with mental health benefits) strains: *L. helveticus* R0052 and *B. longum* R0175. The supplementation of *L. helveticus* R0052 and *B. longum* R0175 lowered anxiety and depression symptoms in research with healthy volunteers. However, no study has studied the effect of this psychobiotic combination on the mental and physical health of MDD patients [78]. Attention-deficit/hyperactivity disorder (ADHD), autism spectrum disorder (AUT), bipolar disorder (BD), schizophrenia (SCZ), and major depressive disorder are examples of frequent mental conditions (MDD). 

Many bacteria and other microbes inhabit the natural environment of the human body. The genomic content of these microorganisms, which surpasses 100 times that of the human genome, is referred to as the microbiome [5]. It has been postulated that disturbed GI microbiota (or dysbiosis) may be harmful in some chronic diseases, with variety, stability, and metabolic activity of the GI microbiota all contributing to health and disease [79].

*Firmicutes*/*Bacteroidetes* ratio, as well as decreases in *Lactobacillus* relative abundance and increases in *Oscillibacter* relative abundance. Although the preclinical research is extensive, only a handful of clinical investigations have explored the microbiota of depressed patients for signs of dysregulation. Reductions in species richness and microbial diversity indicate a dysregulated microbiota in depressed individuals [80].

The most prevalent species in the total microbiome are *Firmicutes*, *Bacteroidetes*, *Actinobacteria*, and *Proteobacteria*. In contrast, the most prevalent bacterial genera are Bacteroides, *Clostridium*, *Peptococcus*, *Bifidobacterium*, *Eubacterium*, *Ruminococcus*, *Faecalibacterium*, and *Peptostreptococcus*, which account for 99 percent [81,82].

*Firmicutes* are dramatically reduced in depressed patients, leading to a decrease in short-chain fatty acids, which may explain the physiological foundation of depression’s low-level inflammation [83]. Numerous species of the genus *Bifidobacterium*, such as *B. adolescentis*, *B. longum*, and *B. dentium*, are elevated in depressive patients, according to one study [84], along with *Lactobacillus* and *Desulfovibrio* species [85].

Bioactive metabolites produced by gut bacteria via the metabolism of dietary tryptophan include indole and its derivatives, such as indole-3-acetic acid (IAA), indole acrylic acid (IA), indole-3-aldehyde (I3A), and indole-3-propionic acid (IPA).

Commensal bacteria use multiple routes to generate and utilize nitric oxide species (NOx). The bacteria in the mouth can turn nitrate (NO_3_) into nitrite with the help of enzymes called nitrate reductases (NO_2_). The chemical conversion of nitrite to nitrate (NO) occurs naturally in the stomach’s acidic environment. Intestinal bacteria can either use respiratory denitrification, dissimilatory and assimilatory nitrate reduction, or both to lower nitrate levels. When nitrate is removed from a solution, it is converted by membrane-bound nitrate reductases into nitrogen oxides (NO and N_2_O) and nitrogen gas (N_2_). This process, known as denitrification, involves the translocation of protons and the production of ATP [86].

### 8.3. Modulation of the Gut–Brain Axis by Probiotics

Probiotics can change the immunological response from T helper (Th2) to Th1. *L. casei* can promote IL-12 production, polarizing the Th1 response and reducing diseases associated with Th2. *L. rhamnosus* inhibits Th2 and Th17 cells and ameliorates the clinical manifestations of seasonal allergies, atopic dermatitis, and psoriatic arthritis. Probiotic fermented dairy milk altered the allergic response induced by ovo-albumin in rats, generating a Th1 rather than a Th2 pattern reaction and resulting in the production of IgG rather than IgE, with elevated levels of IFN- and IL-10 responsible for immunomodulation [46].

Probiotics directly influence the cells of the lamina propria, leading to an increase in the number of IgA-producing cells. IgA plays a crucial role in protecting against mucosal infections, and IgA neutralizes toxins and stops pathogens from attaching to intestinal epithelial cells, preventing disease spread. It has been demonstrated in mice that *L. gasseri* (SBT2055) activates the TLR2 signal pathway, which stimulates IgA-producing cells in the mucosa of the small intestine. While B lymphocytes are primarily responsible for synthesizing immunoglobulin and the adaptive immune response, they can also degrade antibodies by producing IL-10 during inflammatory and chronic illnesses. Using probiotics in conjunction with influenza vaccination boosted an individual’s total number of IgG and memory B-cells [46].

**Figure 6:**
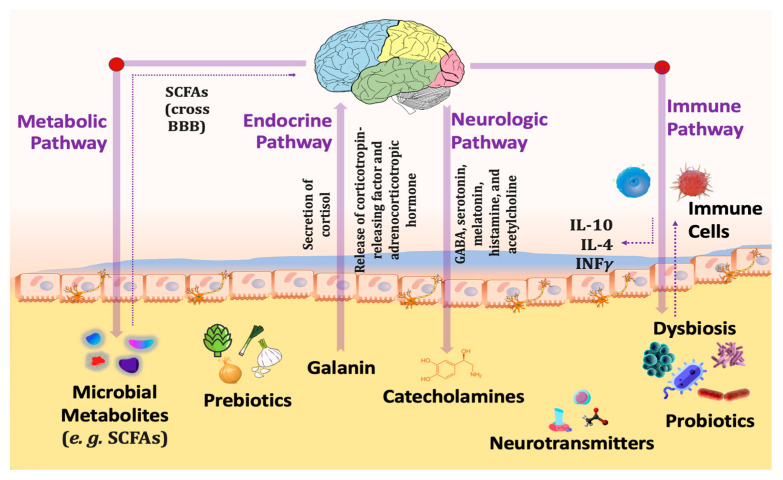
Different pathways connecting gut microbiota with the brain through the gut–brain axis.

The communication pathways (Figure 6) between the gut and the central nervous system involve the vagus nerve, hypothalamic–pituitary–adrenal (HPA) axis, enteric nervous system, endocrine cells, and immune cells [87]. Recent preclinical and clinical research has established that gut microbiota is essential for these gut–brain connections. In addition, abnormalities in the composition of gut microbiota (gut dysbiosis) may contribute to the development of various neurological illnesses, including autism, schizophrenia, depression, Parkinson’s disease, and Alzheimer’s [88].

Increased inflammation, activation of the HPA axis that releases cortisol resulting in intestinal permeability elevation that alters the intestinal microbial profile, altered levels of neurotransmitters including serotonin, dopamine, and glutamate, and bacterial metabolites all contribute to abnormal signaling through the vagus nerve because of gut dysbiosis. Reduced gastrointestinal barrier integrity causes bacterial migration (also known as “leaky gut”) and inflammation. In addition, inflammatory cytokines disrupt the integrity of the blood-brain barrier (BBB), leading to immune cell infiltration, amplifying inflammatory responses, reactive gliosis, and, ultimately, neurodegeneration [88].

Probiotics have the potential to normalize such processes through (1): Reducing stress-induced HPA reactions [89], (2): Decreasing corticosteroid stress reactivity [90], (3): Increasing synthesis of neurotransmitter synthesis such as gamma-aminobutyric acid (GABA), serotonin (5-hydroxytryptamine), dopamine, noradrenaline, melatonin, histamine and acetylcholine, thus influencing mind and behavior [91], (4): Producing SCFAs, primarily acetate, propionate and butyrate, which are essential for gut barrier integrity [92], (5): Enhancing the expression of brain-derived neurotrophic factor (BDNF) that is significant for brain development [93], (6): Stimulating the secretion of gut hormone peptides, such as glucagon-like peptide-1 (GLP-1) [94], and peptide tyrosine (PYY) from enteroendocrine cells [95], (7): Limiting pro-inflammatory cytokine production and inflammation [96], and (8): Triggering the vagal anti-inflammatory reflex, leading to the production of acetylcholine which thereby prevents tissue damage by excessive cytokine release [97].

## 9. Non-Pharmacological Treatments for Psychiatric Disorders

The symptoms of generalized anxiety disorder (GAD) are frequently attributed to physical causes, as indicated by the prevalence of missed and incorrect diagnoses. Successful outcomes may necessitate an individualized combination of treatment modalities. Both psychotherapy and medications, such as selective serotonin reuptake inhibitors, are highly effective in treating depression. Cognitive behavior therapy is well-researched and supported by voluminous evidence among psychotherapeutic interventions [98].

The use of music therapy as an adjunct to non-pharmacological treatments for psychiatric and behavioral disorders is essential. In recent years, it has also been demonstrated that music therapy has a positive effect on individuals with post-traumatic stress disorder who have been exposed to extreme stress. Music therapy is a valuable and underappreciated method of non-pharmacological support for patients with a variety of psychiatric disorders [99]. Meanwhile, Transcranial magnetic stimulation (TMS) is a noninvasive technique for stimulating the brain that has the potential to treat psychiatric disorders. No study has yet examined publication trends in research on TMS modalities for psychiatric disorders, even though several studies have investigated new TMS modalities for the treatment of various psychiatric disorders [100].

## 10. Gut Microbiota and Psychiatric Disorders

### 10.1. Major Depressive Disorder (MDD)

According to the World Health Organization (WHO), major depressive disorder (MDD) will be the most significant cause of disease burden worldwide by 2030 [101]. The monoamine hypothesis was one of the initial hypotheses for the breakdown of neurotransmission in MDD. Numerous antidepressants target the monoamines (serotonin, dopamine, and noradrenaline), which, according to the hypothesis, are profoundly dysregulated in the brains of depressed individuals. 

SCFAs can cross the BBB and stimulate multiple mechanisms in the brain; as a result, SCFAs may stimulate mechanisms possibly involved in the pathobiology of MDD [72].

In young adults, fecal measurements of SCFAs revealed a significant association between acetate, propionate, and butyrate levels and depressive and gastrointestinal symptoms, indicating that SCFAs are significantly linked to the development of MDD [78,102].

Seven studies have investigated the relationship between MDD and human gut flora. Four of these studies focused on the diversity of microorganisms [103], whereas the other three could not find any significant differences in microbial diversity between patients and healthy individuals [104]. Jiang et al. (2015) reported that patients with MDD had a more varied gut microbiome. In a separate investigation, the amount of gut microbiota in individuals with active MDD (A-MDD), responsive MDD (R-MDD), and healthy controls was evaluated [103]. In his study, both A-MDD and R-MDD patients showed a lower abundance of *Firmicutes* than healthy controls, although the number of *Proteobacteria*, *Bacteroidetes*, and *Actinobacteria* increased. However, only A-MDD patients had a greater microbial diversity than R-MDD patients. MDD patients had higher levels of *Alistipes* and *Enterobacteriaceae* and lower levels of *Faecalibacterium* at lower taxonomic levels. In addition, a negative link existed between the severity of depressive symptoms and the *Faecalibacterium* genus.

Another study found a substantial link between *Prevotella* and *Klebsiella* and the severity of depression [105]. This study also revealed that depressed patients had reduced amounts of *Clostridium* XI and *Bacteroidetes* and greater levels of *Firmicutes*, *Prevotella*, *Klebsiella*, and *Streptococcus* [105]. Aizawa et al. (2016) reported that beneficial gut bacteria, such as *Bifidobacterium* and *Lactobacillus*, were similarly reduced in MDD patients [106]. They also showed that the abundance of *Actinobacteria* and *Bacteroidetes* increased while the number of *Firmicutes* decreased in MDD patients, like Jiang’s conclusion. Chen et al. (2021) found that *Firmicutes* and *Actinobacteria* increased in 10 MDD patients compared with 10 healthy controls, whereas *Bacteroidetes* and *Proteobacteria* decreased. Additionally, correlations were found between the degree of depression and the prevalence of *Faecalibacterium* [104].

Painold et al. (2019) identified an association between elevated levels of the phylum *Actinobacteria* and the genus *Faecalibacterium* belonging to *Clostridia* and depression but not MDD. The main species of *Faecalibacterium* is *Faecalibacterium prausnitzii*, and it is known for its role as probiotic bacteria. These bacteria are beneficial against inflammatory bowel disease due to their anti-inflammatory properties [107]. 

However, the evolution of *Bacteroidetes* was not uniform in this research [107]. Current research indicates that MDD patients have fewer *Lactobacilli* and *Firmicutes*, but *Actinobacteria* and *Bacteroidetes* are related to depression. Recent research suggests that the severity and status of MDD patients influence the existence and diversity of microbiota. The reduction and regulation of bacteria that produce short-chain fatty acids facilitate mechanisms that may be implicated in the pathobiology of MDD.

In animal models, many probiotic treatments have demonstrated efficacy in lowering depressive-like behavior. Treatment with a probiotic cocktail containing *L. rhamnosus* and *L. helveticus* strains improved depressive-like behavior and normalized corticosterone levels in an animal model of parental separation. In addition, *L. rhamnosus* therapy reduced depressive and anxious behaviors. There is also evidence of a connection between *Bifidobacterium* strains and animal antidepressant-like behavior. Treatment with a strain of *B. infantis* relieved depression in rats separated from their mothers by enhancing mobility episodes during the forced swim test. Both *B. longum* and *B. breve* strains had a comparable effect on rats’ depression- and anxiety-related behavior [108,109]. 

In addition, researchers have demonstrated that when the microbiota of persons with severe depression is transferred to microbiota-depleted animals, the behavioral and physiological characteristics of depression are also transmitted, establishing a link between dysbiotic microbiota and depression [4]. Certain *Clostridia* species are regarded as helpful members of a healthy gut microbiota and are non-pathogenic. Nevertheless, certain Clostridium species, such as *Clostridium tetani*, *Clostridium botulinum*, and *Clostridium perfringens*, can produce potent toxins known to cause various human illnesses and neurobehavioral symptoms. According to [82], *Sutterella* is a significant component of the intestinal microbiota in more than half of children with autism and GI dysfunction. Still, it is missing in children with only GI dysfunction.

### 10.2. Schizophrenia

As a debilitating mental disease, both positive and negative symptoms characterize schizophrenia, including delusions, hallucinations, and an abnormal thought process (apathy, withdrawal, slowness). It causes severe physical and social morbidity in 21 million people worldwide [110].

Two studies have reported the possibility that abnormalities in the microbiome could serve as biomarkers for schizophrenia. According to one study [111], changes in gamma *proteobacteria* at the class level, *Enterobacteriales* at the order level, and *Bacteroides fragilis* at the species level are associated with the disorder. In contrast, another study discovered that a panel of bacteria from the families *Aerococcaceae*, *Bifidobacteraceae*, *Brucellaceae*, *Pasteurellaceae*, and *Rikenellaceae* is sufficient to differentiate patients from controls [112].

A recent study by Li et al. (2021) reported that the relative abundance of *Ruminococcus* and *Roseburia* was much lower at the genus level in schizophrenia patients compared with controls. However, the abundance of *Veillonella* was significantly higher [113].

Several studies have addressed potential changes and variances in the alpha- and beta-diversity of the microbiome [114]. Beta-diversity represents diversity between groups (i.e., how different was the diversity of bacteria between healthy controls and diseased individuals). In contrast, alpha-diversity depicts diversity within-group (i.e., “how many different species were observed” or “how many different bacteria persist in a healthy individual”), which is generally regarded as a marker of “good” health status [112].

Despite significant differences in beta-diversity between schizophrenia and control groups, most studies have found no differences in alpha-diversity between these groups [115]. This is consistent with the findings of a recent systematic review, which found that beta-diversity was consistently reported to be different between schizophrenia and controls [116].

In a previous study by Nguyen et al. [116], *proteobacteria* were found to be less prevalent in schizophrenia patients, and an earlier study found that chronic schizophrenia patients had a different microbiome beta-diversity than controls, with *proteobacteria* and fusobacteria being significantly more prevalent and firmicutes being significantly less prevalent [111,116]. Ma et al. (2020) and Zheng et al. (2019), who investigated the microbiome in both humans (schizophrenic and controls) and germ-free mice receiving schizophrenia microbiome fecal transplantation, found lower alpha-diversity [112].

Several bacteria (*Aerococcaceae*, *Bifidobacteraceae*, *Brucellaceae*, *Pasteurellaceae*, and *Rikenellaceae*) facilitated the distinction between schizophrenia patients and healthy controls. *Aerococcaceae* and *Rikenellaceae* were the most changed bacterial families in the gut microbiomes of germ-free mice to which researchers transplanted the fecal microbiome of individuals with schizophrenia [114], corresponding to the changes observed in patients with schizophrenia [112].

Butyrate metabolites of SCFAs have been identified to play a function in schizophrenia. Butyrate induces HDAC inhibition, which alters gene expression and plays a role in the epigenetic mark of histone acetylation, which is typically associated with active gene expression [117]. Another study identified high amounts of HDAC1 mRNA and protein in the prefrontal cortex and blood of people with schizophrenia, demonstrating a relationship between HDAC1 overexpression and schizophrenia that can be regulated by butyrate produced by gut microbes [118]. By researching intestinal dysbiosis, it was discovered that the blood-brain barrier and intestinal mucosal barrier were weakened due to a drop in SCFA levels. This demonstrated that a disturbance in gut microbiota leads to microglia-mediated neuroinflammation, which damages neurons, synapses, and the gut–brain axis (GBA). Consequently, these symptoms may be potential causes of the etiopathology of schizophrenia [119].

Indole and its derivatives are significant metabolites produced by gut microbiota, as they play a role in tryptophan metabolism. During dysbiosis, an irregularly elevated tryptophan metabolism was identified, which changes the architecture of white matter in the brains of schizophrenia patients [120]. In rats fed a high-fat diet, indole-3-propionic acid (IPA) altered the makeup of the gut microbiota by preventing microbial dysbiosis and lowering the levels of pro-inflammatory cytokines such as TNF-ɑ, IL-1, and IL-6 [121].

In general, animal studies that have used translationally valid models for schizophrenia have resulted in differing conclusions regarding microbiome changes in schizophrenia; however, there are some points of agreement, such as decreased levels of the phylum *Proteobacteria* and an increase in *Actinobacteria* and *Bacteroidetes* [114].

Probiotic supplementation research in schizophrenia has shed light on the condition. Studies on the effects of probiotic supplements on schizophrenia have yielded contradictory results [122]. In a randomized, controlled trial, giving schizophrenia patients a probiotic supplement comprising Lactobacilli and *Bifidobacterium* bifidum (with vitamin D) decreased CRP levels and increased the plasma’s total antioxidant capacity. This improved the general and complete positive and negative scale syndrome (PANSS) scores, indicating a reduction in inflammation; nevertheless, it remained unclear which factor was responsible for the change [123].

Using the kynurenine pathway, for instance, gut microbiota affects the synthesis of the neurotransmitter 5-hydroxytryptamine (5-HT) in the CNS. By interacting with 5-HT1A and 5-HT2A auto receptors, 5-HT regulates sleep and mood, playing crucial roles in developing insomnia problems and depression. These results imply that some metabolites may mediate the relationship between the brain and the microbiota of the stomach. Nevertheless, the gut microbiota and serum metabolites associated with insomnia are mainly unknown [124]. Consequently, abnormalities of gut microbiota have been demonstrated in numerous psychiatric disorders, such as schizophrenia and bipolar disorder [125], major depressive disorder (MDD) [105], dementia [126], autism, alcoholism, and chronic fatigue syndrome (CFS) [127].

Along with the sympathetic and parasympathetic divisions of the autonomic nervous system (ANS), the enteric nervous system (ENS), and the neuroendocrine and neuroimmune components of the central nervous system (CNS), the gut microbiota is components of a complex network known as the microbiota–gut–brain axis [128].

However, the likely mechanisms of action are not well understood. Probiotics may affect mood via regulating the hypothalamic–pituitary–adrenal (HPA) axis and, in turn, affecting stress management, which in turn improves gut wall integrity and reduces inflammation (including the influence of bacterial-derived metabolites on the microenvironment) [129].

Among these crosstalk pathways, the gut microbiota produces a wide range of bioactive chemicals or metabolites that exert pleiotropic effects on the human organism. Numerous microbial metabolites can penetrate the blood-brain barrier (BBB) or profoundly impact the brain, playing a crucial part in the so-called microbiota–gut–brain axis [130].

Microbes produce several neuroactive chemicals and neurotransmitters, including SCFA, serotonin, gamma-aminobutyric acid (GABA), dopamine, norepinephrine, acetylcholine (Ach), and histamine. In addition, microbiota influence the maturation of microglia, neurogenesis, and the expression of specific neurotransmitter receptors in the central nervous system (CNS) [74].

## 11. Conclusions

The importance of the gut–brain axis in maintaining homeostasis has long been appreciated. Much recent work has implicated the gut microbiota in many conditions, including autism, anxiety, obesity, schizophrenia, Parkinson’s disease, and Alzheimer’s disease. Increased *Enterobacteriaceae* family were potentially associated with a higher risk of schizophrenia; thus, keeping these types of bacteria in balance will help alleviate the adverse effects of other pathogenic bacteria that might cause several diseases. According to the growing body of research, the gut microbiota is becoming crucial in psychiatric disorders. This necessitates further experimental study to deeply describe the complex interaction between the brain and gut microbiota. We believe that advancing the science in this arena will help improve/develop novel psychiatric drugs that help millions of people over the globe.

## Data Availability

All data generated in this word are included within the manuscript.
